# Screening the dermatological potential of *Plectranthus* species components: antioxidant and inhibitory capacities over elastase, collagenase and tyrosinase

**DOI:** 10.1080/14756366.2020.1862099

**Published:** 2020-12-15

**Authors:** Joana M. Andrade, Eva María Domínguez-Martín, Marisa Nicolai, Célia Faustino, Luís Monteiro Rodrigues, Patrícia Rijo

**Affiliations:** a Research Center for Biosciences and Health Technologies (CBIOS), Universidade Lusófona de Humanidades e Tecnologias, Lisboa, Portugal; b Department of Biomedical Sciences, Faculty of Pharmacy, University of Alcalá, Madrid, Spain; c Research Institute for Medicines (iMed.ULisboa), Faculty of Pharmacy, University of Lisbon (ULisboa), Lisbon, Portugal

**Keywords:** *Plectranthus*, antioxidant, tyrosinase, collagenase, elastase

## Abstract

A series of *Plectranthus* spp. plant extracts (aqueous, acetonic, methanolic and ethyl acetic) obtained from eight different species, and previously isolated compounds (ranging from polyphenols, diterpenes and triterpenes), were assayed for *in vitro* inhibition of the skin-related enzymes tyrosinase, collagenase and elastase, and for studying their antioxidant properties. The ethyl acetic extracts of *P. grandidentatus* and *P. ecklonii* registered the highest antioxidant activity, whereas acetonic, methanolic and ethyl acetic extracts of *P. ecklonii*, *P. grandidentatus*, *P. madagascariensis* and *P. saccatus* concerning the enzymatic inhibition assays revealed high anti-tyrosinase and anti-collagenase activities. From the isolated compounds tested, abietane diterpenes and triterpenes were highly active against tyrosinase and elastase activity. Overall, the experimental results showed the powerful antioxidant and inhibitory action on skin-related enzymes tyrosinase, collagenase and elastase of *Plectranthus* spp. extracts and/or isolated compounds, supporting their further research as bioactive metabolites against skin sagging and hyperpigmentation in cosmetic and pharmaceutical formulations.

## Graphical Abstract

**Figure:**
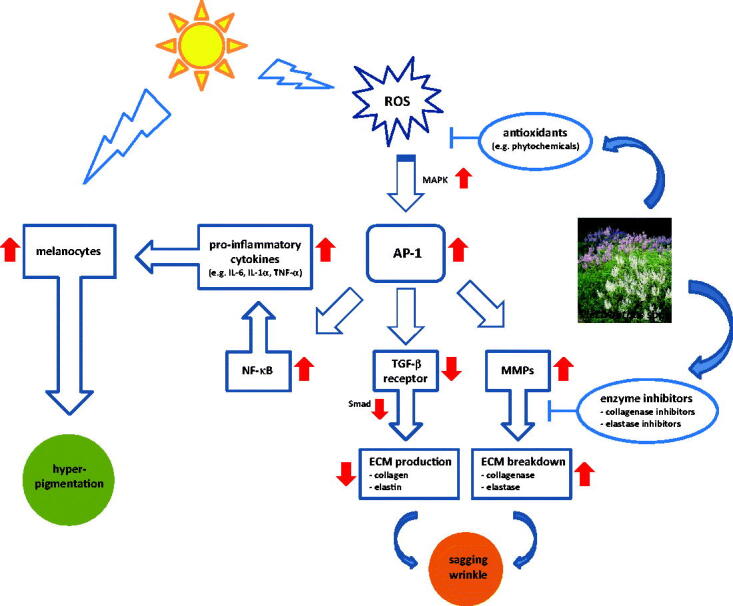

## Introduction

Skin care is a growing concern for the consumer, who is increasingly aware of its maintenance for the preservation of favourable aesthetic appearance, and on its direct impact on the prevention of many skin disorders^1^. In the last years, more attention has been devoted to skin as a mirror for general health condition. Dermatological “links” have been reported to the main cardiovascular pathologies^2–4^, metabolic disease^5^, renal disease^6^, obesity^7^^,^^8^ and, of course, primary ageing^9^^,^^10^. Neurodegenerative diseases such as dementia, Alzheimer’s and Parkinson’s diseases have also been related to dermatological disorders^11^^,^^12^. All these examples illustrate how skin provides new directions in the search of new indicators for diseases prevention, detection, following and treatment.

**Figure 1.**
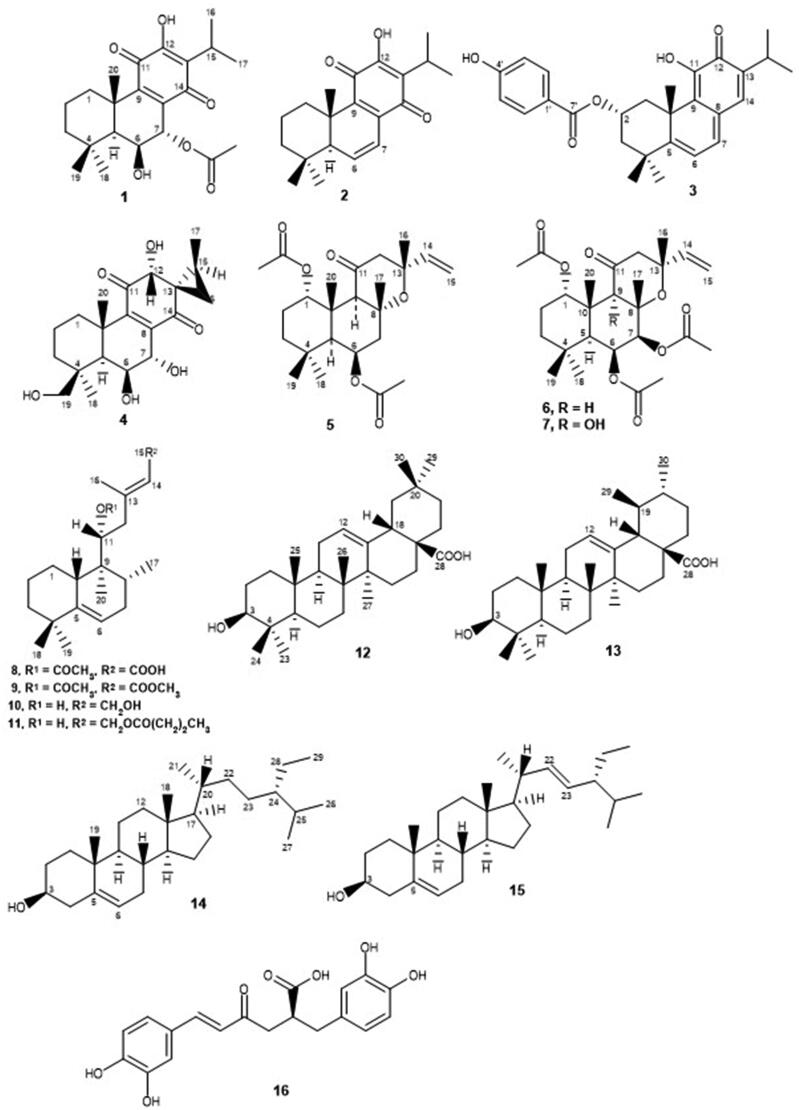
Signalling pathway induced by ultraviolet (UV) radiation causing skin damage. Radical oxygen species (ROS) up-regulated mitogen activated protein kinases (MAPK) cascades enhance transcriptional activity of activator protein 1 (AP-1) heterodimer (comprised of c-Jun and c-Fos) thus increasing metalloproteinases (MPPs) expression. Additionally, transforming growth factor (TGF)-/Smad signalling pathway is down-regulated by over-accumulation of ROS, decreasing the synthesis of extracellular matrix (ECM) proteins such as collagenase and elastin.

The origin of many of these skin disorders has been linked to oxidative stress^13^^,^^14^, which is commonly promoted by chemicals, microorganisms and ultraviolet (UV) solar radiation^15–17^. Exposure to UV radiation can be particularly adverse for people with low melanin production, such as Caucasians, due to the fact of generating reactive oxygen species (ROS), such as superoxide anions, hydrogen peroxide (H_2_O_2_) and hydroxyl radicals. ROS can react with cellular lipids, deoxyribonucleic acid (DNA) and proteins to evoke lipid peroxidation, enzyme denaturation, mutagenesis and eventually cell death^16^^,^^18^. One of the determinants of extrinsic ageing processes are these oxidative reactions, which are catalysed in the presence of redox-active metal ions like Fe^2+^ and Cu^2+^, commonly found in the biological environment. All these factors, particularly UV, can modify the extracellular matrix (ECM) breaking down structural proteins such as collagen and elastin (Figure 1), which are the major components of the dermal tissue, essential for skin’s structural stability and biomechanics^17^^,^^19^. ROS have been shown to upregulate the expression of several proteinases, including matrix metalloproteinases (MMP) and serine proteases, such as collagenase and elastase^14^. They were also reported to be involved in chronic inflammatory skin diseases, neurodegenerative disorders as well as in extrinsic ageing^9^^,^^17^^,^^20^.

The search for new and more effective molecules from different sources has clearly been focussed on these potential mechanisms, with the goal of preventing oxidation, thus reducing the production of ROS and protecting the tissues^14^^,^^21^. Plants from the genus *Plectranthus*, belonging to the *Lamiaceae* family, are widely distributed across the warm and tropical areas of Africa, Asia and Oceania, and were likely brought to Mediterranean areas in the 16th century during the period of Portuguese discoveries^22^^,^^23^. Rich in essential oils and in mono- and sesquiterpenes^24–26^, extracts from these plants are widely used in traditional medicine with different ethnopharmacological uses, including antimicrobial, analgesic, antipyretic, anti-inflammatory and antitumoral. Moreover, they have been applied in many skin disorders including wound healing^25–28^. Diterpenoids (abietane, labdane, kaurane, and clerodane skeleton), together with triterpenoids, phytosterols, phenolic acids, flavonoids and other polyphenolic compounds are the specific compounds of interest within this genus, according to their displayed effects compiled in the literature^28–30^. Polyphenols, such as rosmarinic acid which is ubiquous to all *Plectranthus* spp., and chlorogenic acid, obtained primarily from *P. saccatus*, and usually present in the aqueous extracts^31–34^, are powerful antioxidants^17^^,^^22^. Phenol groups in polyphenolic compounds are able to accept an electron to form relatively stable phenoxyl radicals, thereby disrupting radical chain oxidation reactions in cellular components^35^. Abietane diterpenes from *P. ecklonii*, *P. madagascariensis* and *P. grandidentatus* are able to chelate the 2,2-diphenyl-1-picrylhydrazyl (DPPH) radical comparably to positive control quercetin, a natural flavonoid ^23^^,^^33^.

Another consequence of ROS overproduction on Caucasian skin is hyperpigmentation, frequently leading to lentigine lesions, freckles and melanoma^21^^,^^36^. All of these result from the stimulation of tyrosinase, a binuclear copper rate-limiting oxidase acting in the biosynthesis of melanin from L-tyrosine^21^^,^^36–38^. Overall, the chelating ability of *Plectranthus* spp., notably of *P. ecklonii*, *P. madagascariensis* and *P. grandidentatus* secondary metabolites, seems to be useful in a synergy for both antioxidant and anti-pigmentation skin treatment.

The degradation of the dermal ECM is also associated with these phenomena, such as the reduction or inhibition of collagenase and/or elastase activities, which might contribute to their prevention^17^^,^^39^. Recent studies have reported that many phenolic compounds isolated from plants with antioxidant properties, such as catechin and epigallocatechin gallate (EGCG), are also collagenase and/or elastase inhibitors. Inhibition usually involves metal chelation, making the catalytic Zn^2+^ of collagenase unavailable^19^^,^^40^. The hydrogen bonding between the hydroxyl groups of polyphenols and hydrogen bond donor or acceptor groups from elastase domains, hydrophobic interactions between the benzene rings of polyphenols and hydrophobic functional groups of the enzymes, and induced conformational changes^17^^,^^37^. Triterpenoids common to many plants, including some *Plectranthus* spp. such as *P. neochilus, P. ornatus* and *P. ecklonii*, are strong elastase and collagenase inhibitors^20^^,^^36^^,^^40–42^; they can bind reversibly to the catalytic sites of the enzymes, since an activity recovery has been observed upon dilution of the enzyme-inhibitor mixture^43^.

**Figure 2.**
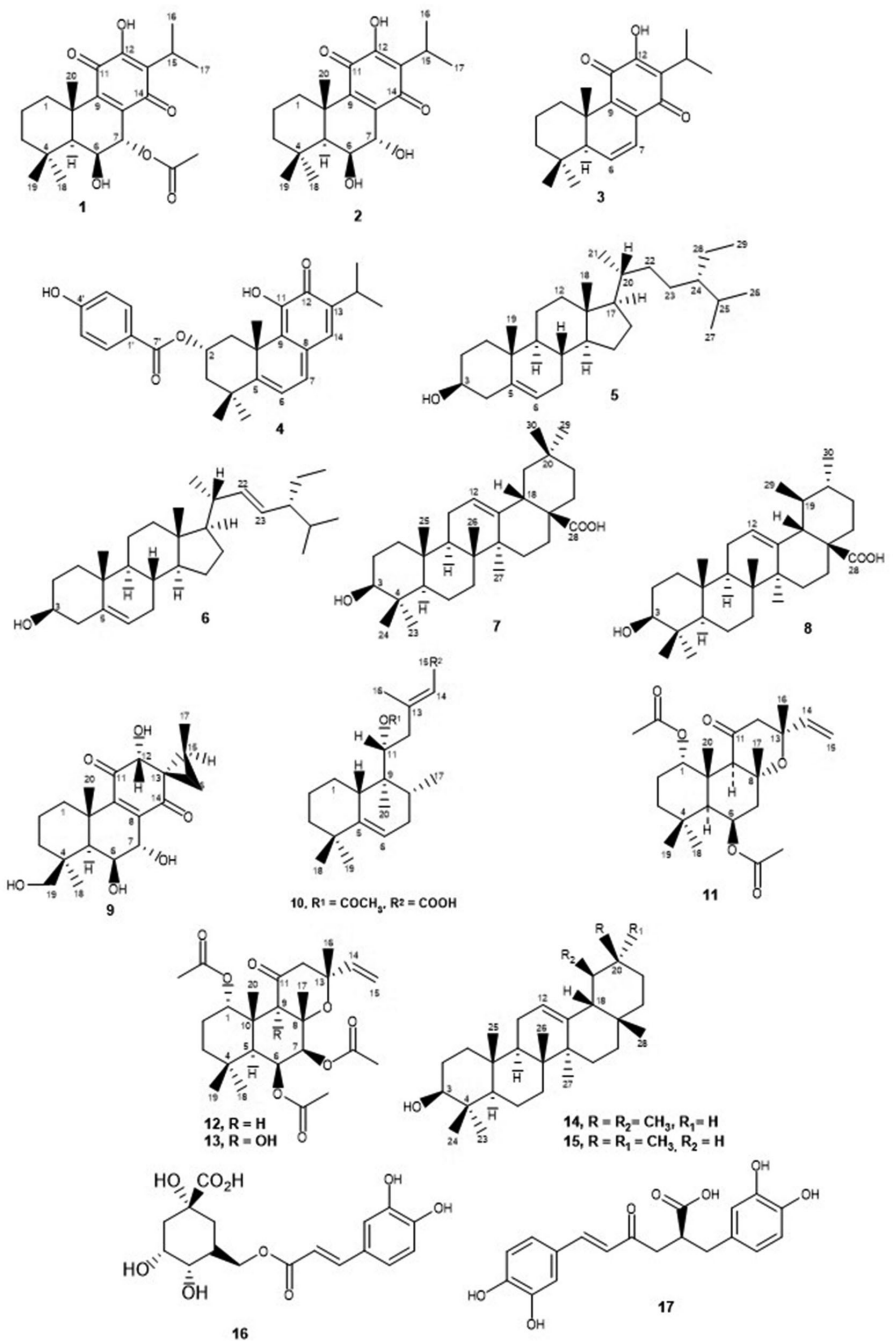
Studied natural compounds isolated from *Plectranthus* spp: 7-acetoxy-6-hydroxyroyleanone (**1**), 6,7-dihydroxyroyleanone (**2**), 6,7-dehydroroyleanone (**3**), Parvifloron D (**4**), -sitosterol (**5**), stigmasterol (**6**), oleanolic acid (**7**), ursolic acid (**8**), (13*S*,15*S*)-6,7,12,19-tetrahydroxy-13,16-cyclo-8-abietene-11,14-dione (**9**), (11 R*,13*E*)-11-acetoxyhalima-5,13-dien-15-oic acid (**10**), Plectrornatin C (**11**), 1,6-di-*O*-acetylforskolin (**12**), 1,6-di-*O*-acetyl-9-deoxyforskolin (**13**), -amyrin (**14**), -amyrin (**15**), chlorogenic acid (**16**) and rosmarinic acid (**17**).

This study represents a primary evaluation of *Plectranthus* spp. extracts and isolated compounds as potential antioxidant agents capable of inhibiting the skin-related enzymes tyrosinase, collagenase and elastase, for the synergistic treatment of skin disorders, namely ageing, sagging and hyperpigmentation of the skin. Organic and aqueous extracts of several *Plectranthus* spp. plants, as well as their isolated compounds (Figure 2) were obtained, characterised and screened by our team, following the procedures described in previous phytochemical studies^23^^,^^30–34^^,^^44–46^. Their antioxidant and specific anti-enzymatic activities were tested in support of their potential interest for application in preventive dermatology and/or synergistic therapeutics. Furthermore, this is the first report on the *in vitro* activity of *Plectranthus* spp. extracts and isolated compounds as tyrosinase, collagenase and elastase inhibitors.

## Materials and methods

### Reagents and enzymes

2,2-Diphenyl-1-picrylhydrazyl (DPPH), quercetin, L-tyrosine, kojic acid, epigallocatechin gallate (EGCG), ursolic acid, *N*-[3-furyl-acryloyl]-Leu-Gly-Pro-Ala (FALGPA), *N*-succinyl-Ala-Ala-*p*-nitroanilide (SANA) and phosphate-buffered saline (PBS) were from Sigma-Aldrich. Tris(hydroxymethyl) aminomethane (Tris base buffer) was from Prolabo and tricine buffer was from Amresco. The enzyme elastase (EC 3.4.21.36) from porcine pancreas was purchased to Alfa Aesar, while tyrosinase (EC 1.14.18.1) from mushroom and collagenase (EC 3.4.24.3) from *Clostridium histolyticum* type IA were obtained from Sigma-Aldrich. Methanol, ethanol 70% (w/v), dimethyl sulfoxide (DMSO), ethyl acetate and acetone were obtained from Merck.

### Plant material, extract preparation, and isolated compounds

*Plectranthus* (*P. grandidentatus* Gürke, *P. ecklonii* Benth., *P. ornatus* Codd., *P. madagascariensis* (Pers.) Benth., *P. porcatus* van Jaarsv. & P.J.D. Winter, *P. neochilus* Schltr. and *P. prostratus* Gürke) medicinal plants from South Africa were cultivated at “Instituto Superior de Agronomia” campus (Lisbon). Extraction methods were performed according to previously established literature procedures, with slight modifications^44^.

The organic extracts were obtained from 10 0.010 g of air-dried aerial parts and powdered plants in 200 ml of methanol, ethyl acetate or acetone after sonication at room temperature for 1 h. The organic extracts were filtered and the solvent was removed in a rotary evaporator at 40–50 °C. The crude extracts (21 samples) were stored at 20 mg/mL in DMSO.

The aqueous extracts were obtained from 10 0.010 g of the aerial parts of dried and powdered plants in 150 ml of bi-distilled water (Milli-Q) after microwave-assisted extraction for 3 min at a continuous irradiation of 2.45 GHz. The aqueous extracts (7 samples) were filtered and separated into 1 ml aliquots (in triplicate) for freeze-drying and frozen at −20 °C. After freeze-drying, the extracts were weighted and stored at 10 mg/mL in bi-distilled water (Milli-Q) at −20 °C. The amount (dry weight) of each plant extract and yields according to solvent and extraction methodology are shown in Table 1.

**Table 1.** Amount of *Plectranthus* spp. extracts and yields according to the extraction solvent and extraction method (MW, microwave; US, ultrasound).

*Plectranthus* spp.	Solvent (Method)	Dry residue/g	Yield %
		(0.001 g)	(mg/100g)
*P. grandidentatus*	Water (MW)	0.028	0.27
	Acetone (US)	0.237	2.37
	Methanol (US)	0.686	6.76
	Ethyl acetate (US)	0.247	2.45
*P. madagascariensis*	Water (MW)	0.031	0.31
	Acetone (US)	0.203	2.03
	Methanol (US)	0.823	8.21
	Ethyl acetate (US)	0.236	2.35
*P. ecklonii*	Water (MW)	0.034	0.34
	Acetone (US)	0.384	3.83
	Methanol (US)	0.949	9.45
	Ethyl acetate (US)	0.332	3.32
*P. porcatus*	Water (MW)	0.026	0.26
	Acetone (US)	0.662	6.59
	Methanol (US)	1.233	12.30
	Ethyl acetate (US)	0.704	7.00
*P. ornatus*	Water (MW)	0.043	0.43
	Acetone (US)	6.000	59.90
	Methanol (US)	1.059	10.60
	Ethyl acetate (US)	0.924	9.15
*P. neochilus*	Water (MW)	0.032	0.32
	Acetone (US)	0.430	4.26
	Methanol (US)	1.040	10.40
	Ethyl acetate (US)	0.552	5.51
*P. prostratus*	Water (MW)	0.033	0.33
	Acetone (US)	0.819	8.16
	Methanol (US)	1.189	11.80
	Ethyl acetate (US)	0.951	9.48
*P. saccatus*	Acetone (US)	0.689	6.89

The natural compounds tested (Figure 2), previously isolated from *Plectranthus* spp. using bioassay-guided fractionation of extracts, were the abietane diterpenoids 7-acetoxy-6-hydroxyroyleanone (**1**) from *P. grandidentatus*^34^^,^^46^, 6,7-dihydroxyroyleanone (**2**) and 6,7-dehydroroyleanone (**3**) from *P. madagascariensis*^22^, Parvifloron D (**4**), the phytosterol 1:1 mixture of -sitosterol (**5**) and stigmasterol (**6**), and the 4:1 mixture of oleanolic (**7**) and ursolic acids (**8**), from *P. ecklonii*^31^^,^^46^, (13*S*,15*S*)-6,7,12,19-tetrahydroxy-13,16-cyclo-8-abietene-11,14-dione (**9**) from *P. porcatus*^32^, the halimane diterpene (11 R*,13*E*)-11-acetoxyhalima-5,13-dien-15-oic acid (**10**), the labdane diterpenoids Plectrornatin C (**11**) and the 1:1 mixture of 1,6-di-*O*-acetylforskolin (**12**) and 1,6-di-*O*-acetyl-9-deoxyforskolin (**13**), the triterpene 3:1 mixture of -amyrin (**14**) and -amyrin (**15**) from *P. ornatus*^30^^,^^33^^,^^45^^,^^47^, chlorogenic acid (**16**) from *P. saccatus*^22^ and rosmarinic acid (**17**), the main polyphenol isolated from the aqueous extracts of *Plectranthus* spp.^44^. The chemical structures of these compounds have been established by comparing their spectral data with those in the literature and/or with authentic samples isolated by our group.

### Antioxidant activity assay

The free radical scavenging activity of the organic extracts (100 g/mL) was evaluated by the DPPH assay according to literature procedures^33^^,^^48^. All extracts were dissolved in ethanol 70% (w/v), mixed with DPPH solution (100 mM in ethanol) and incubated at room temperature for 30 min, in the dark. The absorbance of the solutions was read at 517 nm (Perkin-Elmer Lambda 2 UV-vis spectrophotometer) against a blank containing the same concentration of the organic extracts in ethanol. Quercetin (IC_50_ = 10.3 1.5 g/mL) was used as positive control while negative control corresponded to DPPH in ethanol. Radical scavenging activity (RSA, %) was determined from Equation (1).

(1) $$\text{RSA}\;\left(\%\right)\;={\;\text{Abs}}_{\left(\text{DPPH}\right)}–\;\left[\left({\text{Abs}}_{\left(\text{sample}\right)}–{\;\text{Abs}}_{\left(\text{blank}\right)}\right)/{\text{Abs}}_{\left(\text{DPPH}\right)}\right]\;100$$

### Enzyme inhibitory assays

#### Anti-tyrosinase activity assay

The isolated compounds (Figure 2) and extracts (both organic and aqueous) from the described *Plectranthus* plants were tested in the anti-tyrosinase activity assay with modifications^15^. The assay was performed using 180 L of the substrate L-tyrosine (0.5 mM) in PBS 50 mM (pH 6.8) and 10 L of the tested samples (50 g/mL) incubated for 5 min at 37 C before starting the reaction by adding 10 L of tyrosinase (5000 U). After incubation at 37 C for 5 min, production of dopachrome was detected from absorbance measurements at 450 nm every 2 min, for 10 min, in a microplate reader (Thermo-Fisher Scientific). Kojic acid (0.8 mM) was used as positive control, with reported IC_50_ of 43.7 M^49^, and sample solvent (DMSO 0.5% (v/v) in PBS buffer) as negative control. All assays were performed in triplicate. Results were expressed as percentage inhibition (%) determined from Equations (2) and (3). The absorbance variation (Abs) registered by Equation (2) for enzyme velocity reaction of negative control (Abs/time) must be in the linear range.

(2) Velocity reaction of control or inhibitor = Abs/time (min)

(3) $$\text{Inhibitory activity }\left(\%\right)\;=100\;–\;\left(100{\text{ velocity reaction}}_{\left(\text{inhibitor}\right)}\right)/{\text{velocity reaction}}_{\left(\text{control}\right)}$$

#### Anti-collagenase activity assay

The anti-collagenase enzymatic assay was optimised based on several methods reported in the literature^16^^,^^17^^,^^40^^,^^50^. The synthetic substrate *N*-[3-furyl-acryloyl]-Leu-Gly-Pro-Ala (FALGPA) 0.1 mM was dissolved in tricine buffer 50 mM (pH 7.5) supplemented with 400 mM sodium chloride (NaCl) and 10 mM calcium chloride (CaCl_2_) (assay buffer). Collagenase was prepared in the assay buffer at 1 U, knowing that 1 U hydrolyses 1 mol of FALGPA per minute, at 25 °C, in the presence of calcium ions. EGCG at 40 M was used as positive control with reported IC_50_ of 0.9 mM^51^, and the sample solvent (DMSO 0.3% v/v in tricine buffer) as the negative control. The assay mixture containing 80 L of tested samples (100 g/mL) and 100 L of collagenase was incubated at 37 °C for 10 min before starting the reaction by adding 20 L of FALGPA. The assay was performed in triplicate. Absorbance of FALGPA was read at 405 nm for 10 min, continuously, in a microplate reader (Thermo-Fisher Scientific). Results were expressed as percentage inhibition (%), according to Equations (2) and (3).

#### Anti-elastase activity assay

The anti-elastase enzymatic assay was based on spectrophotometric methods described in the literature, with some modifications^16^^,^^17^^,^^19^^,^^52^. The substrate *N*-succinyl-Ala-Ala-Ala-*p*-nitroanilide (SANA) 1 mM was dissolved in Tris-HCl buffer 50 mM (pH 8.0), knowing that 1 U enzyme (elastase) converts 1 mol of SANA per minute in this buffer at 25 °C. Ursolic acid (100 g/mL) was used as positive control with reported IC_50_ of 10 M^19^, and the sample solvent (DMSO 1% (v/v) in Tris-HCl buffer) as negative control. The reaction was initiated by adding 20 L of SANA and 150 L of Tris-HCl buffer followed by addition of elastase (6 U) and the samples (100 g/mL). The mixture was incubated at 25 °C for 10 min. Formation of *p*-nitroaniline from SANA hydrolysis was detected from absorbance measurements at 405 nm, performed immediately after starting the reaction and then every 30 s for 3 min, in a microplate reader (Thermo-Fisher Scientific). All assays were performed in triplicate. Results were expressed as percentage inhibition (%) according to Equations (2) and (3).

### Statistical analysis

Data comparisons were conducted with one-way analysis of variance (ANOVA) followed by *post-hoc* Tukey honest significant difference test, for pairwise comparisons. Analysis and graphical presentation were performed with the GraphPad Prism Software Version 5 (GraphPad Software, Inc., San Diego, CA, USA). Values of *p* < 0.05 were statistically significant. Results were presented as mean standard deviation (SD).

## Results

### Antioxidant activity

**Figure 3.**
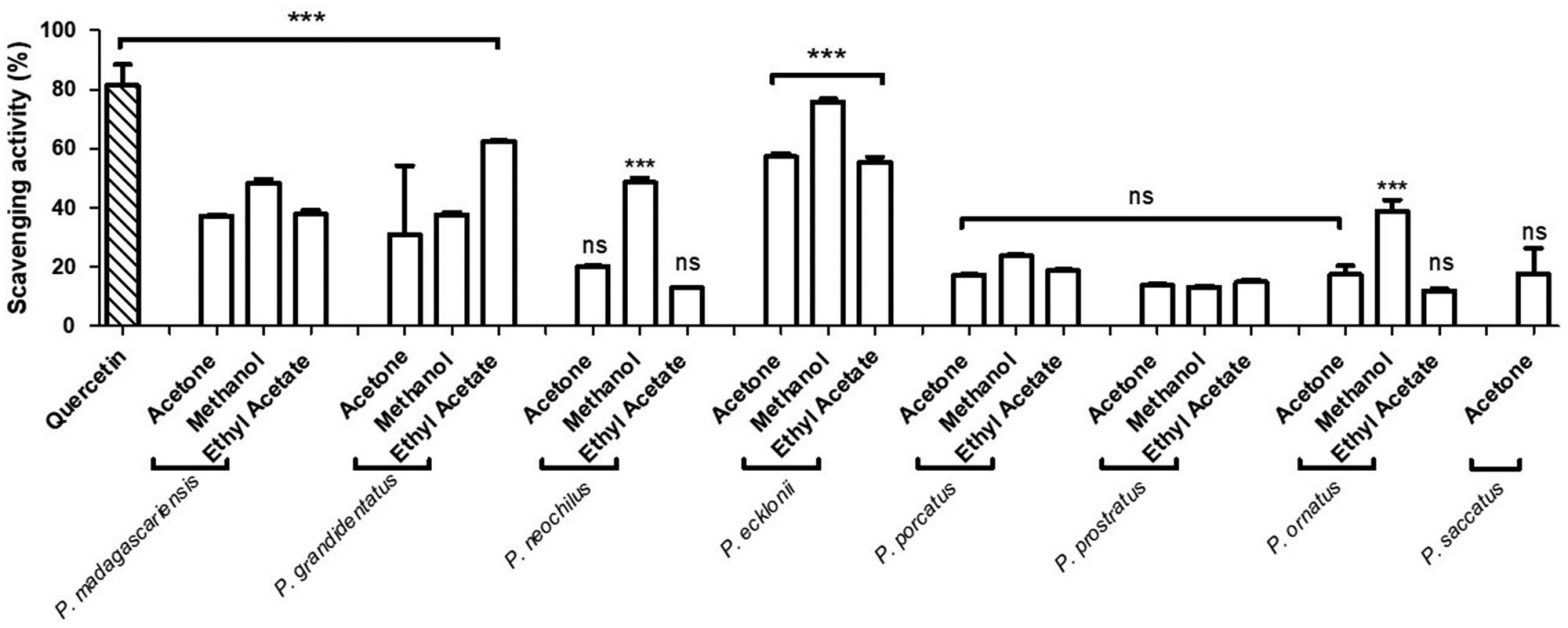
*In vitro* antioxidant activity of *Plectranthus* spp. organic extracts at 100 g/mL measured as percentage of DPPH radical scavenging activity. The results are presented as means percentage values, considering the absorbance of quercetin as the positive control. Data are expressed as the mean ± SD (*n* = 3) **p < 0.005 ***p < 0.0001 *vs* negative control (DPPH in ethanol). DPPH: 2,2-diphenyl-1-picrylhydrazyl; ns: not significant; SD: Standard Desviation. Values were determined by one-way ANOVA followed by Tukey HSD comparison test.

The antioxidant activity of several *Plectranthus* spp. organic extracts was evaluated concerning their ability for scavenging the DPPH radical, using quercetin as positive control. The results, expressed in percentage of RSA, are shown in Figure 3.

### Inhibition of skin-related enzymes

#### In vitro tyrosinase inhibition

The present study performed a screening of several *Plectrathus* spp. extracts (both aqueous and organic) and previously isolated secondary metabolites regarding their *in vitro* inhibition of skin-related enzymes, namely tyrosinase, collagenase and elastase.

**Figure 4.**
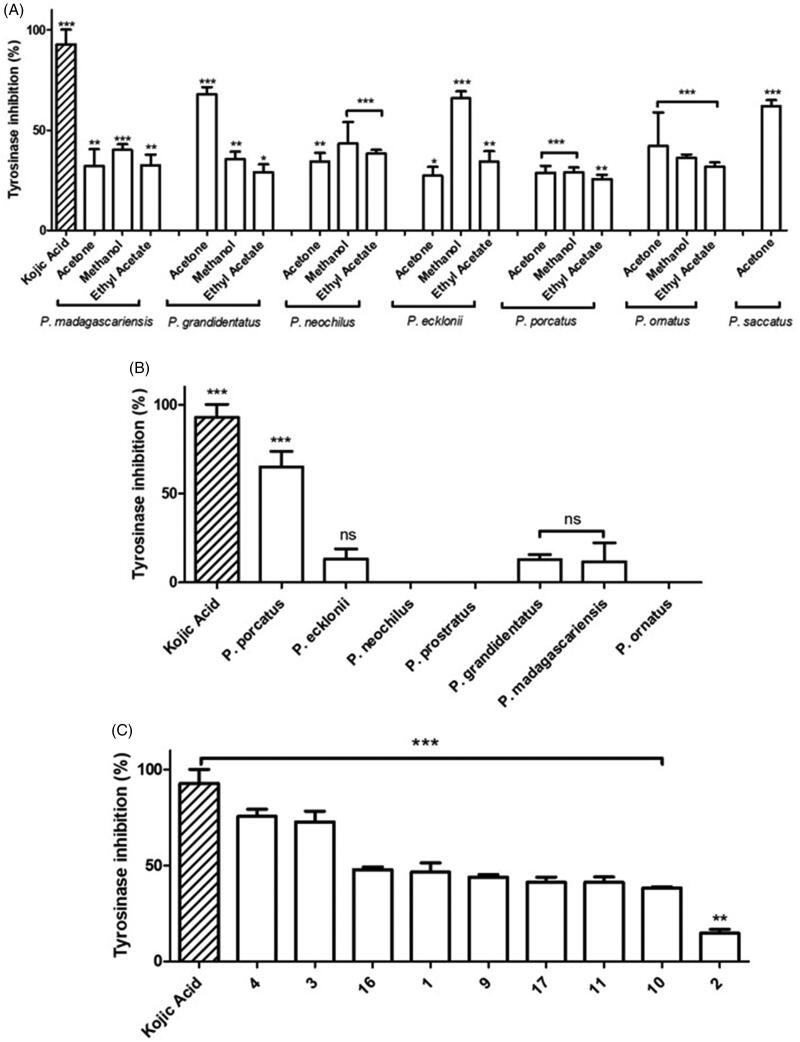
*In vitro* anti-tyrosinase activity of *Plectranthus* spp. (A) Organic extracts at 50 g/mL. (B) Aqueous extracts at 50 g/mL. (C) Isolated compounds at 50 g/mL. The results are presented as means percentage values, considering the absorbance of kojic acid as the positive control. Data are expressed as the mean ± SD (*n* = 3) *p < 0.05 **p < 0.005 ***p < 0.0001 vs negative control (DMSO 0.5% (v/v) in PBS buffer). DMSO: dimethyl sulfoxide; ns: not significant; PBS: phosphate-buffered saline; SD: Standard Deviation. Values were determined by one-way ANOVA followed by Tukey HSD comparison test.

In the *in vitro* anti-tyrosinase assay, the enzymatic activity was evaluated by using L-tyrosine as substrate and detecting the produced chromophore (dopachrome) at 450 nm^49^ as described in Materials and Methods - *Anti-tyrosinase Activity Assay*. The results obtained for the *Plectranthus* spp. organic extracts shown in Figure 4(A), expressed as percentage of inhibition of tyrosinase activity, ranged from 25 to 68% compared to 92.9 7.4% obtained for kojic acid, used as positive control.

Aiming at a more extensive comprehension of the agents causing tyrosinase inhibition, the aqueous extracts obtained from *Plectranthus* spp. were additionally tested, and the results obtained, presented in Figure 4(B), revealed that in contrast with the organic extracts, the aqueous extracts were less effective as tyrosinase inhibitors.

To better understand the observed results, an additional assay was performed to evaluate which natural products present in the organic and aqueous extracts could be responsible for the exhibited anti-tyrosinase activity. Thus, previously isolated compounds from both organic and aqueous extracts of *Plectranthus* spp. were tested for their ability to inhibit tyrosinase *in vitro*, and the results obtained are shown in Figure 4(C).

#### *In vitro* collagenase inhibition

**Figure 5.**
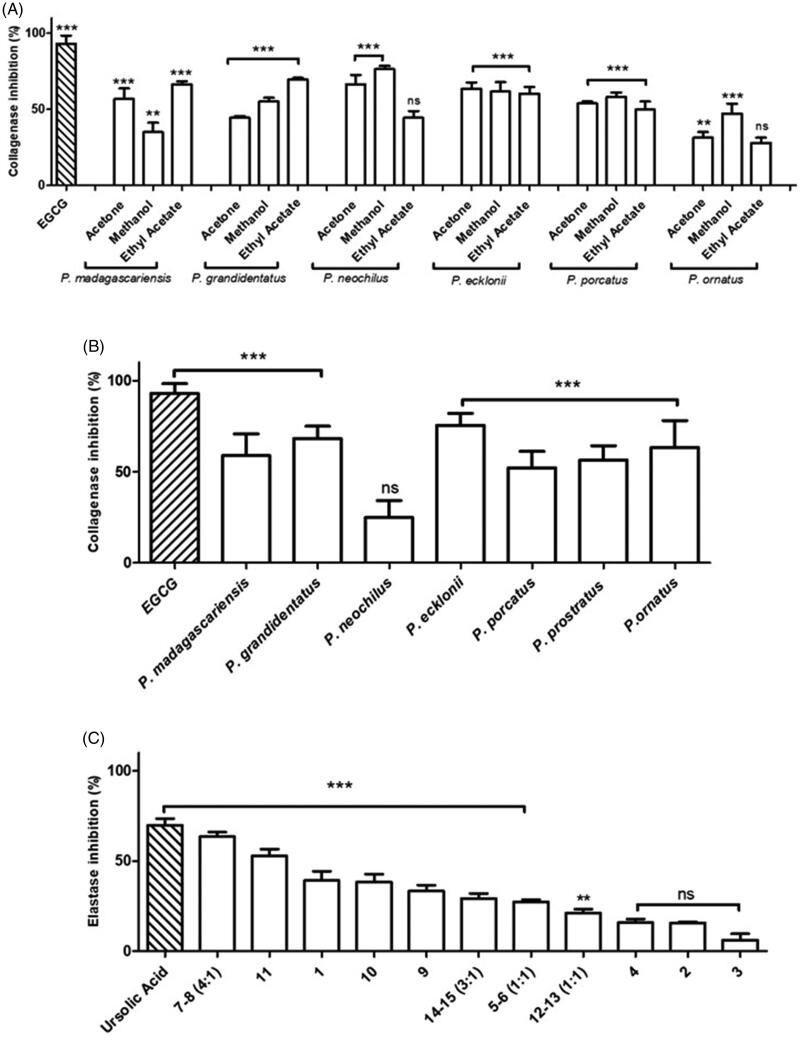
*In vitro* anti-collagenase activity of *Plectranthus* spp. (A) Organic extracts at 100 g/mL. (B) Aqueous extracts at 100 g/mL. (C) Isolated compounds at 100 g/mL. The results are presented as means percentage values, considering the absorbance of EGCG as the positive control. Data are expressed as the mean ± SD (*n* = 3) ***p < 0.0001 *vs* negative control (DMSO 0.3% (v/v) in Tricine buffer). DMSO: dimethyl sulphoxide; EGCG: epigallocatechin gallate; ns: not significant; SD: Standard Deviation. Values were determined by one-way ANOVA followed by Tukey HSD comparison test.

Evaluation of the *in vitro* anti-collagenase activity of *Plectranthus* spp. extracts and isolated compounds was performed using FALGPA as substrate and measuring the collagenase (ChC) activity by the decrease in absorbance at 405 nm due to FALGPA hydrolysis as described in Materials and Methods – *Anti-collagenase Activity Assay*. Figure 5(A) represents the results obtained for the organic extracts, expressed as percentage inhibition, revealing a mild to high ChC inhibitory activity, ranging from 28% to 76%, compared with EGCG used as positive control.

The aqueous extracts from *Plectranthus* spp., characterised by high amounts of phenolic compounds, which have been described as collagenase inhibitors^14^, were also studied for ChC inhibition, and results are displayed in Figure 5(B).

Since both organic and aqueous extracts from *Plectranthus* spp. in general exerted an inhibitory effect on collagenase, isolated compounds were further tested in order to determine their contribution for the enzymatic inhibition observed, and results obtained are exhibited in Figure 5(C).

#### *In vitro* elastase inhibition

**Figure 6.**
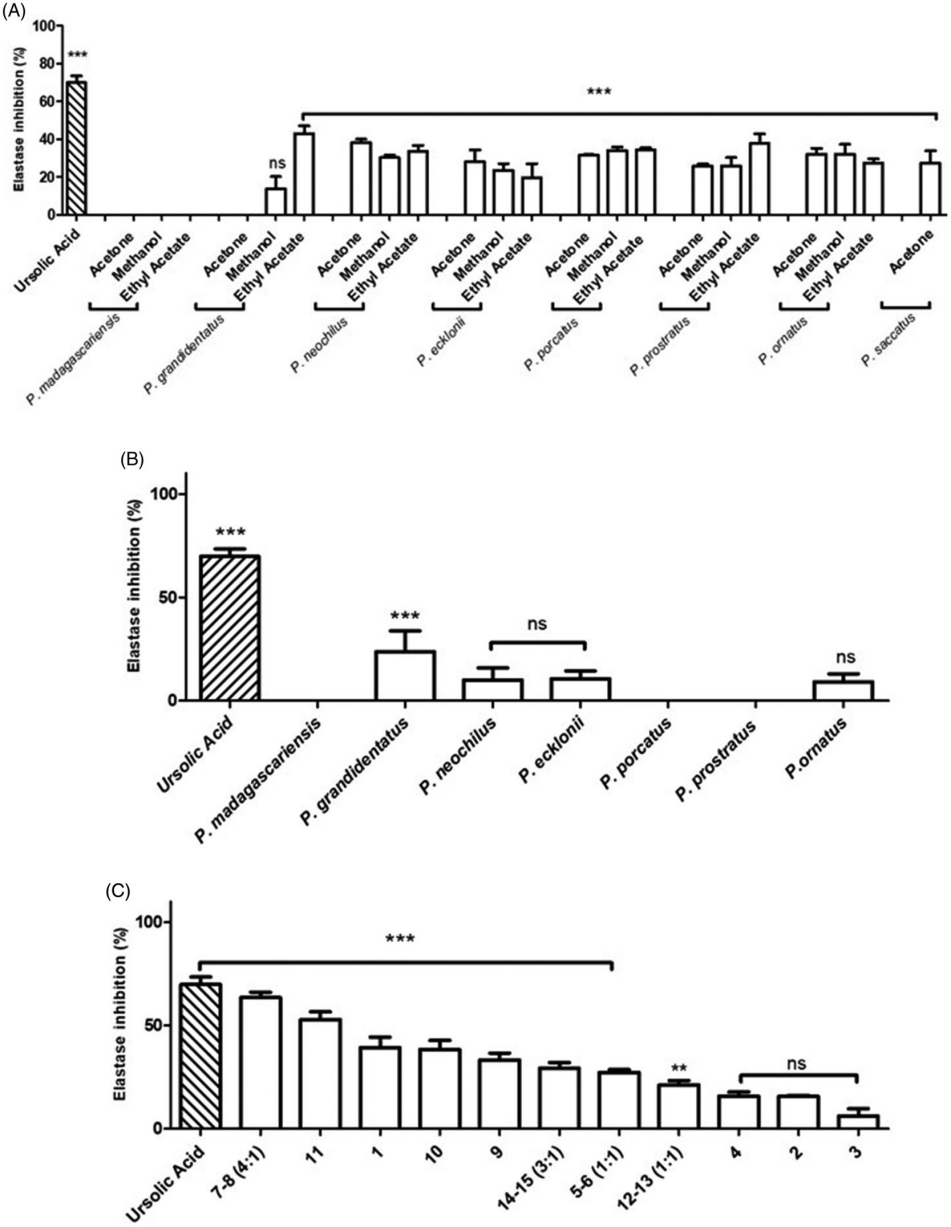
*In vitro* anti-elastase activity of *Plectranthus* spp. (A) Organic extracts at 100 g/mL. (B) Aqueous extracts at 100 g/mL. (C) Isolated compounds at 100 g/mL. The results are presented as means percentage values, considering the absorbance of ursolic acid as the positive control. Data are expressed as the mean ± SD (*n* = 3) ***p < 0.0001 vs negative control (DMSO 1% (v/v) in Tris-HCl buffer). DMSO: dimethyl sulphoxide; HCl: Hydrochloride; ns: not significant; SD: Standard Deviation; Tris: tris(hydroxymethyl) aminomethane. Values were determined by one-way ANOVA followed by Tukey HSD comparison test.

The anti-elastase activity of *Plectranthus* spp. extracts and isolated compounds was evaluated *in vitro* using SANA as substrate and detecting the formation of *p*-nitroaniline at 405 nm resulting from SANA hydrolysis, as described in the Materials and Methods - *Anti-elastase Activity Assay*, using ursolic acid as positive control. Results of the enzymatic assay concerning the organic extracts from *Plectranthus* spp. plants are expressed in Figure 6(A), revealing in general very weak elastase inhibitory activity.

Since polyphenolic compounds have been suggested to possess anti-elastase activity^20^, the aqueous extracts expected to have this type of compounds were tested and the results are shown in Figure 6(B).

Although aqueous extracts from *Plectranthus* spp. plants were also not effective as elastase inhibitors (*p* > 0.05), according to Figure 5(B), the isolated compounds were still further assessed for their ability to inhibit elastase and results are displayed in Figure 6(C).

## Discussion

### Antioxidant activity

The antioxidant properties of *Plectranthus* spp. organic extracts were evaluated based on their scavenging activity for the DPPH radical. According to Figure 3, the methanolic extracts held the highest RSA (20–76%) among the organic extracts, except for the ethyl acetic extract from *P. grandidentatus*, with a RSA of 62.3 0.4%. The observed results are possibly related with the high content of polyphenols usually present in the methanolic extracts, which are known for their antioxidant activity^53^. Nevertheless, recent findings concerning the antioxidant activity of abietane diterpenes, namely compounds (**1**) and (**4**)^23^, which have been isolated from *Plectranthus* spp., suggest that these compounds may also contribute to the observed bioactivity. The quinone moiety present in abietane diterpenes is probably responsible for their biological activity, since quinones represent important features at stabilising free radicals in many biological systems suffering from oxidative stress, protein inactivation and intermediate melanin synthesis pathway in human skin^54^^,^^55^.

The antioxidant results obtained for the methanolic extracts of *P. ecklonii* (75.9 1.0%) and *P. madagascariensis* (48.4 1.2%) as well as for the ethyl acetic extract of *P. grandidentatus* (62.3 0.4%) can also be explained by their main diterpenoid compounds with an abietane backbone associated with antioxidant activity^23^. Additionally, *P. ecklonii* ethyl acetic (55.5 1.7%) and acetonic (56.9 1.5%) extracts showed relatively higher RSA when compared to the remaining *Plectranthus* spp. extracts, which held high RSA values only for the methanolic extracts. Actually, *P. ecklonii* and *P. grandidentatus* had the highest antioxidant activity among the studied *Plectranthus* species, with RSA similar to that of quercetin (89.0 2.5%) used as positive control, which is a potent antioxidant flavonoid compound^23^^,^^33^.

Recently there has been an increased concern about synthetic antioxidants, such as the widely used butylated hydroxyanisole (BHA) and butylated hydroxytoluene (BHT), which are suspected to induce liver damage and carcinogenesis in animals^39^. Therefore, less cytotoxic and eventually more powerful antioxidants from natural sources, such as *Plectranthus* plants, represent promising alternatives.

### *In vitro* inhibition of skin-related enzymes

#### Tyrosinase inhibition

The *Plectranthus* spp. extracts and isolated compounds have been evaluated as tyrosinase inhibitors. The organic extracts showed promising results (Figure 4(A)) with inhibitory activity as high as 68% compared to 92.9 7.4% obtained for kojic acid used as positive control. According to the results obtained (Figure 4(A)), the methanolic (25–66%) and acetonic (27–68%) extracts exhibited the highest tyrosinase inhibition activity (*p* < 0.05). High tyrosinase inhibition was observed for the *P. grandidentatus* acetonic (67.9 3.6%), *P. ecklonii* methanolic (65.9 3.4%) and *P. saccatus* acetonic (56.4 5.7%) extracts. Moreover, *P. grandidentatus* and *P. ecklonii* showed the highest anti-tyrosinase activity *in vitro*, and these were also the plants that showed increased antioxidant activity. The observed results can be attributed to the abietane diterpenoids mainly present in the organic extracts of these two plants^23^^,^^33^.

In contrast with the organic extracts, the aqueous extracts (Figure 4(B)) were less effective as tyrosinase inhibitors (*p* > 0.05), except for the aqueous extract of *P. porcatus* (*p* < 0.0001), showing tyrosinase inhibition of 65.0 8.7% in comparison with 92.9 7.4% obtained for kojic acid used as positive control. Actually, the *P. porcatus* aqueous extracts obtained with microwave extraction have been previously characterised by high-performance liquid chromatography (HPLC) concerning quantification of polyphenols^22^. The obtained HPLC profile revealed that *P. porcatus* aqueous extracts have some amounts of rosmarinic acid (**17**) and caffeic acid, however *P. ecklonii* and *P. saccatus* had the highest polyphenol content^22^. Therefore, it can only be suggested that the anti-tyrosinase activity of the aqueous extract of *P. porcatus* is probably due to a synergistic effect of the present compounds.

Further evaluation of the effects on tyrosinase activity of isolated compounds from both organic and aqueous extracts (Figure 4(C)) confirm some of the high inhibition values previously found for the extracts. Notably, the abietane diterpenes (**1**), (**3**) and (**4**) present mainly in *P. grandidentatus*, *P. madagascariensis*, and *P. ecklonii* organic extracts, are able to inhibit tyrosinase activity in more than 46% and up to 75%. Moreover, it is possible to better understand the activity of the *P. porcatus* aqueous extract according to the result obtained for compound (**9**) which inhibited tyrosinase activity by 43.9 1.3%. Although this compound is mainly isolated from the acetone extract of *P. porcatus*, which showed low tyrosinase inhibition (28.8 3.4%, Figure 4(A)), its presence in the aqueous extract cannot be excluded since the microwave aqueous extraction method has a higher efficiency in the recovery of bioactive compounds^22^. Additionally, (**16**) and (**17**) were able to inhibit tyrosinase by 47.9 1.4% and 40.4 0.7%, respectively. However, these compounds are not present in high amounts in *P. porcatus* aqueous extracts, therefore it can only be suggested that the extract activity was probably due to the presence of compound (**9**). Overall, the results suggest that *Plectranthus* spp. polyphenols and abietane diterpenes are capable of inhibiting tyrosinase being almost as efficient as kojic acid used as positive control.

Although neither the mechanism of inhibition nor the type of inhibition were studied, the most potent tyrosinase inhibitors such as hydroquinone, kojic acid, azelaic acid and other electron-rich phenols, have been reported for their capability of inhibiting melanin overproduction^21^^,^^37^. However, the use of these compounds in the fight against skin ageing and hyperpigmentation is limited due to their adverse side effects, low formulation stability, and poor skin penetration, hence the search for new agents from natural products.

Hydroquinones and phenols, part of the chemical structure of abietane diterpenes (**1**–**4**), (**16**) and (**17**), have been recognised for their chelating ability^21^, which can probably explain their high anti-tyrosinase activity. Moreover, due to the presence of polyphenolic compounds such as chlorogenic acid (**16**) and quercetin, other plants have been used for the treatment of skin depigmentation^14^. Actually, quercetin, a strong antioxidant flavonoid compound, has been reported to be a strong inhibitor of tyrosinase, with an IC_50_ of 0.10 mM^37^. This strongly suggests that the observed synergistic effect of *Plectranthus* spp. natural products with both antioxidant and anti-tyrosinase inhibitor properties can be useful for anti-pigmentation skin treatment.

#### Collagenase inhibition

The organic extracts from *Plectranthus* spp. exhibited mild to high ChC inhibitory activity, in the range 28–76% (Figure 5(A)). The highest anti-collagenase activity was observed for *P. neochilus* methanolic extract (76.4 2.1%) in comparison with the positive control EGCG (93.1 5.3%). Additionally, the organic extracts of *P. madagascariensis*, *P. grandidentatus*, and *P. ecklonii* were effective at inhibiting ChC in more than 60%.

The obtained results for the *P. neochilus* organic extracts strongly suggest that the anti-ChC activity observed is mainly due to pentacyclic triterpenes typically present, particularly (**14**) and (**15**), previously reported as ChC inhibitors^43^. On the other hand, the high inhibition of *P. ecklonii* organic extracts is most likely due to the presence of other pentacyclic triterpenes, such as (**7**) and (**8**) and/or abietane diterpenoids (**1**–**4**). Actually, compounds (**7**), (**8**), (**14**) and (**15**) have been widely studied for their ability to inhibit both collagenase and elastase, possibly by reversibly binding to the catalytic sites of these enzymes^19^^,^^36^^,^^43^.

Phenolic compounds have also been described as collagenase inhibitors^14^, thus the aqueous extracts from *Plectranthus* spp. containing the larger amounts of these compounds were also evaluated for ChC inhibition (Figure 5(B)). In contrast with the organic extracts, the *P. neochilus* aqueous extract was the lowest ChC inhibitor (24.9 9.4%), although the remaining aqueous extracts revealed more promising results, the highest inhibition (75.6 6.5%) being obtained for the *P. ecklonii* aqueous extract.

Previous studies concerning polyphenols quantification in *Plectranthus* spp. plants have established that *P. ecklonii* aqueous extracts obtained from microwave extraction had one of the highest contents on compound (**17**)^34^. This study helps to understand the high collagenase activity observed for the *P. ecklonii* aqueous extract since many polyphenolic compounds, such as catechin and EGCG, have been reported to inhibit collagenase^40^, probably by acting as metal chelators, making the Zn^2+^ ion unavailable for catalytic activity^19^^,^^40^.

In general, both organic and aqueous extracts from *Plectranthus* spp. exerted an inhibitory effect on collagenase, thus isolated compounds were further tested (Figure 5(C) in order to determine their contribution for the enzymatic inhibition observed. According to Figure 5(C), the diterpene abietanes (**3**) and (**4**) mainly isolated from organic extracts along with (**17**), the major polyphenol present in the aqueous extracts, showed the highest ability for collagenase inhibition. Notably, compound (**4**) was found to inhibit ChC by 84.6 5.9%, being almost as efficient as positive control EGCG (93.1 5.3%). To our knowledge, this is the first report on the *in vitro* inhibition of ChC by diterpenes with an abietane backbone.

On the other hand, the royleanones (**1**) and (**2**) inhibited ChC by only 33.5 3.3% and 24.0 3.0%, respectively. These compounds are also abietane diterpenes, but with a royleanone motif that may be lowering the inhibitory capacity when compared to (**4**), whose structure includes different donor atoms resulting in higher metal chelation ability^23^. Compound (**17**) inhibited collagenase by 44.8 4.5% suggesting the presence of other polyphenols, such as compound (**16**) or caffeic acid, in the aqueous extracts of *Plectranthus* spp. to possibly justify the reported activity in Figure 5(B). Overall, *Plectranthus* spp. plants are highly promising natural sources for developing potential cosmetic agents against skin ageing induced by increased collagenase activity.

#### Elastase inhibition

The anti-elastase activity of *Plectranthus* spp. organic extracts (Figure 6(A)) revealed impaired elastase inhibition, in contrast to the previous enzymatic assays regarding tyrosinase and collagenase inhibitory activity. Under the experimental conditions, the maximum elastase inhibition obtained was 42.8 4.2% for *P. grandidentatus* ethyl acetic extract, while the positive control, ursolic acid, inhibited elastase by 69.9 3.7%. The organic extracts from *P. madagascariensis* and the acetonic extract from *P. grandidentatus* showed no elastase inhibition (*p* > 0.05).

Mild elastase inhibition (around 30%) was observed for the organic extracts from *P. neochilus* and *P. ecklonii*, probably due to the presence of different types of triterpenes, such as compounds (**7**), (**8**), (**14**) and (**15**). Moreover, previous studies suggested that polyphenolic compounds may have anti-elastase activity due to interaction of the hydroxyl groups with the elastase domain ^20^. Therefore, the *Plectranthus* spp. aqueous extracts expected to have this type of compounds, were further evaluated for anti-elastase activity (Figure 6(B)).

The results on Figure 6(B) reveal that the aqueous extracts from *Plectranthus* spp. plants were not effective as elastase inhibitors (*p* > 0.05), being the *P. grandidentatus* aqueous extract the only one able to inhibit the elastase enzyme, by 23.8 10.0% (*p* < 0.0001). This result can be attributed to different and decreased synergy of the polyphenols present in *P. grandidentatus* aqueous extract, in comparison with the remaining aqueous extracts^34^. Nevertheless, the isolated compounds from both organic and aqueous extracts were further assessed for their ability to inhibit elastase activity (Figure 6(C)).

In marked contrast to the *Plectranthus* extracts, the isolated compounds were strong elastase inhibitors. In accordance with the positive control, ursolic acid, compounds (**7**) and (**8**) in 4:1 mixture showed a high anti-elastase activity of 63.4 2.7% (*p* < 0.0001). These preliminary results strengthen the literature reports regarding the anti-elastase activity of the pentacyclic triterpenes^20^^,^^36^^,^^40–42^. On the other hand, compounds with pentacyclic triterpene structure similar to that of compound (**8**), such as (**14**) and (**15**), which were anticipated to have high anti-elastase activity according to previous studies^40^, showed no significant activity. This may be due to interaction on elastase possibly involving different subdomains^20^.

Besides the triterpenes, royleanone-like diterpenes were also very effective in elastase inhibition. These compounds, including (**4**) (52.8 3.8%), (**2**) (39.2 5.2%) and (**3**) (38.3 4.4%), showed highest elastase inhibitory activity (Figure 6(C)). Thus, elastase inhibitors obtained from *Plectranthus* spp. plants are possible candidates for the treatment or prevention of skin photoageing.

The results obtained with the isolated compounds from *Plectranthus* spp. plants concerning the *in vitro* enzyme inhibitory assays of the three skin-related enzymes (tyrosinase, collagenase and elastase), which are summarised in Table 2, suggest that *Plectranthus* spp. natural products are promising bioactive agents for future formulations against hyperpigmentation, wrinkle, and sagging of the skin.

**Table 2.** *In vitro* enzymatic inhibition (%) of tyrosinase, collagenase and elastase by isolated compounds from *Plectranthus* spp.

	Enzymatic inhibition SD (%)		
Compound No.	Anti-tyrosinase^a^	Anti-collagenase^b^	Anti-elastase^b^
1	46.6 4.7	33.5 3.2	29.3 2.8
2	14.7 2.0	24.0 3.0	39.2 5.2
3	75.7 3.6	60.6 9.7	38.3 4.4
4	72.7 5.6	84.6 5.9	52.8 3.8
5-6 (1:1 mixture)	NT	NT	NT
7-8 (1:4 mixture)	NT	NT	63.5 2.6
9	43.9 1.3	24.6 9.2	21.2 2.1
10	38.3 0.6	41.5 4.9	27.3 1.4
11	41.3 2.7	16.7 1.9	33.3 3.3
12-13 (1:1 mixture)	NT	NT	6.15 3.5
14-15 (3:1 mixture)	NT	NT	15.8 2.0
16	40.4 0.7	NT	NT
17	47.9 1.4	44.8 4.5	NT
Positive control (compound)	92.9 7.4 (Kojic acid)	93.1 5.3 (EGCG)	69.9 3.7 (Ursolic acid)

^a^Samples tested at 50 g/mL, ^b^samples tested at 100 g/mL, NT, not tested; for compound number chemical structure assignments refer to Table 1. EGCG: epigallocatechin gallate.

The experimental results showed that the methanolic extracts from *P. ecklonii* and *P. madagascariensis*, as well as the *P. grandidentatus* ethyl acetic extract, generally had the highest values for radical scavenging activity and enzymatic inhibition. Interestingly, the isolated compounds mainly present in these plant extracts (abietane diterpenes, triterpenes, and polyphenols) also revealed high inhibitory effects on tyrosinase, collagenase and elastase enzymes. The highest inhibitory activity of *P. porcatus* aqueous extract in the anti-tyrosinase assay, which contains only minor amounts of polyphenolic compounds, is probably due to the presence of the diterpenoid (**9**). Despite the lower efficiency observed in the elastase inhibition assay, the abietane diterpene (**4**) showed high inhibitory activity only surmounted by the triterpene mixture of (**7**–**8**).

In summary, all the plants had antioxidant properties, with methanolic extract having the highest scavenging activity compared to other organic extracts. The organic extracts showed promising results. *P. grandidentatus* acetonic extract, *P. eckolonii* methanolic extract and *P. saccatus* acetonic extract showed high tyrosinase inhibition. The aqueous extracts were not good inhibitors, except for *P. porcatus* extract which was better than the other plants extracts. The isolated compounds (**1**), (**3**) and (**4**), from both organic and aqueous extracts, were able to inhibit tyrosinase. The organic extract showed high collagenase inhibition, the highest anti-collagenase activity was observed for *P. neochilus* methanolic extract. For the aqueous extracts, *P. ecklonii* showed the highest inhibition. Compounds (**3**), (**4**) and (**17**) also showed good inhibition.

The organic extracts were not good inhibitors of the elastase enzyme, the percentage inhibition was too low compare to the positive control. *P. grandidentatus* ethyl acetic extract have some activity but it was low. The aqueous extracts were also not good inhibitors of elastase, again *P. grandidentatus* was better than all the other plants extracts but it was not good.

Thus, due to their antioxidant properties and *in vitro* ability to inhibit the skin-related enzymes (tyrosinase, collagenase, and elastase), the *Plectranthus* spp. extracts and isolated compounds represent promising bioactive agents with strong potential in cosmetic and/or pharmaceutical formulations to be developed against wrinkling, photo-ageing, hyperpigmentation and sagging of the skin. With this objective in mind, an article has recently been published that studies two different basic formulations containing *P. ecklonii* extracts, one in an organic solvent and the other using water^56^.

Recent discoveries on non-neuronal cholinergic system as a regulator of skin (patho)physiology have drawn attention to the importance of acetylcholinesterase (AChE) in the human skin^57^^,^^58^. Apart from the extensively known role of AChE in the termination of nerve impulse transmission at the cholinergic synapses by rapid hydrolysis of the neurotransmitter acethylcholine, the rationale underlying the use of AChE inhibitors in the symptomatic treatment of Alzheimer’s disease^59^^,^^60^. Thus, further studies concerning anti-AChE activity *in vitro* will be conducted based on previous reports of high AChE inhibition by some *Plectrathus* spp. aqueous extracts (*P. ecklonii*, *P. grandidentatus*, *P. ornatus*, *P. porcatus* and *P. saccatus*) attributed to the presence of rosmarinic (**17**) and caffeic acids as major compounds^14^^,^^34^^,^^44^^,^^61^^,^^62^.
